# Health and well-being in metal musicians: a scoping review

**DOI:** 10.3389/fpsyg.2026.1737492

**Published:** 2026-04-13

**Authors:** Céleste Rousseau, Dirk Möller, Alice Vincent, Corentin Charbonnier

**Affiliations:** 1 Department of Movement and Rehabilitation Science, Faculty of Business, Management, and Social Science, University of Applied Sciences Osnabrück, Osnabrück, Germany; 2 Unité de Recherche UR 20201 REHADAPT, Université Versailles Saint-Quentin – Paris Saclay, Versailles, France; 3 USR 3608 République des savoirs, École Normale Supérieure, Paris, France; 4 Independent Researcher, Paris, France; 5 UMR 7324 CITERES, Université de Tours, Tours, France

**Keywords:** metal music, musicians’ health, performance, popular music, prevention

## Abstract

**Introduction:**

Elite musicians face high risks of performance-related health problems, yet research mainly targets classical players. Metal musicians, despite unique demands (fast tempos, headbanging, extreme vocal techniques) remain under-investigated, highlighting urgent need for broader, inclusive studies on musicians’ health.

**Methods:**

This scoping review, guided by methodological recommendations, maps existing evidence on metal musicians’ health worldwide. It identifies gaps across physical, and mental well-being, using systematic searches, inclusion criteria, and structured data analysis.

**Results:**

From more than 5,000 references, 47 studies were included, and then categorised into musculoskeletal, vocal, hearing, mental health, mortality, and other issues. Findings highlight specific risks for metal/rock musicians, including injuries, hearing loss, vocal strain, psychological challenges, and elevated mortality rates.

**Discussion:**

This first scoping review synthesises evidence on metal musicians’ health, highlighting musculoskeletal risks, vocal resilience, hearing loss, psychological stress, and elevated mortality. Despite shared issues with other musicians, unique practices like headbanging demand targeted research, prevention, and culturally informed approaches.

## Introduction

Playing a musical instrument at an elite level requires significant commitment, both physically and mentally, leading to a high prevalence of performance-related health problems (PRHPs) among musicians, regardless of level (amateur, student, professional) or repertoire among other characteristics (Kok et al., 2016; Silva et al., 2015). These health issues encompass various disorders, including musculoskeletal (Kok et al., 2016) and neurological (Altenmüller and Jabusch, 2010), auditory (Di Stadio et al., 2018), vocal (Pestana et al., 2017), and mental health problems (Kenny et al., 2014). Consequently, as music is now considered a high-risk occupation (Chan and Ackermann, 2014), musicians’ health has become a growing area of interest, reflected in an increasing of publications and the development of dedicated health services, research laboratories, and associations worldwide.

While research into musicians’ health is becoming increasingly prevalent, the populations studied remain mostly homogeneous. In their systematic review focusing on musculoskeletal complaints in musicians, Kok et al. (2016) noted the underrepresentation of “non-classical musicians,” such as marching band members or pop/rock musicians. A few years later, Stanhope and Weinstein (2020) published a narrative review entitled “Why do we need to investigate non-classical musicians to reduce the burden of musicians’ musculoskeletal symptoms?”, highlighting substantial differences in posture, movement, technique, repertoire, and approaches to prevention or management of PRHPs between classical and non-classical musicians, exemplified through comparisons between classical violin and Irish fiddle players.

Among under-represented musicians in literature, metal musicians are particularly notable. Heavy metal, a subgenre of rock music, emerged in the early 1970s, primarily in the United Kingdom and the United States of America, with Americans favouring Led Zeppelin and the British favouring Black Sabbath (Weinstein, 2000). The genre peaked in popularity in the 1980s, propelled by British bands such as Iron Maiden or Def Leppard (Kahn-Harris, 2007), and through periods of recognition beyond the metal community (Charbonnier, 2024). While metal is known as a musical genre, it is also considered as a distinct culture, with its own codes, values, myths, and rituals (Charbonnier, 2015). It has sometimes been reductively referred as “heavy metal,” first subgenre before referring all to *metal* (especially in American literature), though the genre now comprises more than one hundred subgenres, with diverse instrumentation and no single unifying history. While metal music may have “polished its coat of arms” and gained broader recognition—such as the performance of Gojira at the Paris 2024 Olympic Games—metal musicians themselves remain under-investigated, despite likely facing similar, or even unique, PRHPs, particularly during touring and with advancing age.

While some risks factors for metal musicians may align with those already investigated in other genres, metal presents specific features. For example, drummers and guitarists often play at extremely high tempos (Xhignesse, 2024), potentially leading to specific conditions, such as the case of lower limb task-specific focal dystonia in a metal drummer (Lee and Altenmuller, 2014). Metal-specific forms of movements, or dances also warrant attention (Charbonnier, 2018), and particularly headbanging, mentioned in the aforementioned review (Stanhope and Weinstein, 2020) and considered in case reports (Egnor et al., 1991). In addition to musculoskeletal health, vocal health among metal singers is also of concern, especially due to techniques such as growling and screaming (Guzman et al., 2019; Caffier et al., 2018). Furthermore, auditory health may be compromised by prolonged exposure to loud volumes. Finally, mental health among metal musicians should be thoroughly explored, considering both stereotypes and the diverse psychological profiles that may arise across subgenres and cultural contexts (Charbonnier, 2024 in Charbonnier and Garcin, 2024).

## Objectives

This study aims to investigate both physical and mental health among metal musicians, and also punk and more generally rock musicians—as the literature is particularly sparse on this topic and because these musicians may often share the stage with metal musicians—who may experience distinct performance-related health issues compared to musicians playing classical or other popular music repertoires. Moreover, all of these musicians’ repertoires require high-volume performances, use of instrument effect (such as distortion) or high-speed instrument-related movements, use of voice effect, and presence of specific stage movements.

This research work seeks to address two main questions:

(1) What is the prevalence of physical and mental health disorders among metal musicians?
(2) What are the main potential risk factors for developing physical injuries (musculoskeletal, vocal, hearing, etc.) or mental health conditions among metal musicians?

## Methods

### Methodological decision

Scoping reviews are valuable tools for “rapidly map[ing] the key concepts underpinning a research area and the main sources and types of evidence available” (Arksey and O'Malley, 2005). They are particularly useful for identifying gaps in the literature and laying the groundwork for further research (Arksey and O'Malley, 2005). In the context of metal musicians’ health, available evidence appears limited and fragmented (i.e., many studies mention metal musicians without focusing specifically on them). This situation supports the need for a broader literature review that aggregates evidence across various sources. According to the six principles proposed to choose scoping reviews (Munn et al., 2018; Peters et al., 2020), three factors guided the choice of a scoping review approach: the need to identify types of available evidence, the characteristics and key factors of metal musicians’ health, and the identification of research gaps. This scoping review also serves as a precursor to a larger clinical research project addressing the identified gaps (Munn et al., 2018; Peters et al., 2020).

### Main objectives

Following the Population, Concept, and Context (PCC) framework (Peters et al., 2022):

- Population: Musicians playing metal, rock and punk repertoires
- Concept: Physical, mental and social health and well-being
- Context: Global scope (metal musicians worldwide)

Sub-genres of metal were identified as they were in the *Metallum Musicae Galaxia* developed by Charbonnier (Charbonnier, 2024 in Charbonnier and Garcin, 2024).

### Protocol

The proposed scoping review was conducted in accordance with the framework from Arksey and O'Malley (2005), the Joanna Briggs Institute (JBI) methodology for scoping reviews (Peters et al., 2022; Pollock et al., 2023), and the Preferred Reporting Items for Systematic Reviews and Meta-Analyses extension for Scoping Reviews (PRISMA-ScR) guidelines (Tricco et al., 2018).

#### Search strategy

The search for relevant publications was conducted in three stages. Firstly, an initial electronic search was performed in the following databases: PubMed, Science Direct, Scopus, EBSCO and LILACS.

Search equations included combination of the following terms:

1. Music, musician, instrumentalist, vocalist, singer
2. Metal, heavy metal, rock, hard-rock, punk
3. Health, injury, pathology, muscle, voice

Equations varied from one database to another, due to their specific requirements, and all are listed in Table 1.

**Table 1:** Specific search equations for each database.

Databases	Search equations
PubMed	(metal OR heavy metal OR rock OR hard-rock OR punk) AND (music OR musician OR instrumental* OR vocal* OR singer*) AND (health OR injur* OR patholog* OR muscle OR voice)
Science Direct (Title, Abstract, Keywords)	musician AND (metal OR heavy metal OR rock OR hard-rock OR punk) AND (injury OR pathology)
Scopus (Title, Abstract, Keywords)	(metal OR heavy metal OR rock OR hard-rock OR punk) AND (music OR musician OR instrumental* OR vocal* OR singer*) AND (health OR injur* OR patholog* OR muscle OR voice)
EBSCO (Title, Abstract, Keywords)	(metal OR heavy metal OR rock OR hard-rock OR punk) AND (music OR musician OR instrumental* OR vocal* OR singer*) AND (health OR injur* OR patholog* OR muscle OR voice)
LILACS (Title, Abstract, Subject)	(metal OR heavy metal OR rock OR hard-rock OR punk) AND (music OR musician OR instrumental* OR vocal* OR singer*) AND (health OR injur* OR patholog* OR muscle OR voice)

Secondly, duplicates were removed, and inclusion criteria applied, reference lists of included studies were manually screened. Finally, a manual search was performed using Google Scholar, the bibliography of the International Society for Metal Music Studies, and the specialised journal Medical Problems of Performing Artists.

This search was conducted between 12th August and 19th August 2025, analysed between 30th August and 10th September 2025.

#### Study selection

After duplicate removal, the titles and abstracts of all identified citations were independently analysed by two reviewers (C. R. and A. V.; Arksey and O'Malley, 2005; Peters et al., 2022) applying the following inclusion and non-inclusion criteria to guide the selection process (see Table 2). A pilot screening was conducted, as recommended in the latest JBI guidelines (Peters et al., 2022), on 5% (*n* = 178) of the identified sources at first stage. The screening of 178 potential sources was independently performed before a Zoom-meeting between both reviewers, to compare their inclusion and adapt their screening for the 95% remaining articles.

**Table 2:** Inclusion and non-inclusion criteria according to the PCC framework (Peters et al., 2022).

PCC framework	Inclusion	Non-inclusion
Population	Metal, rock and punk musicians (Figure 1)	Other repertoire musicians, metal music listeners only or general population
Concepts	All studies investigating health components	Studies not investigating health components
Publication type	Peer-reviewed articles (quantitative, qualitative or mix-methods studies, as well as reviews) Editorials Conference abstracts Grey literature, such as unpublished theses or dissertations, professional journals articles	Books
Language	English, French, German, Italian	All other languages except those included

**Figure 1:**
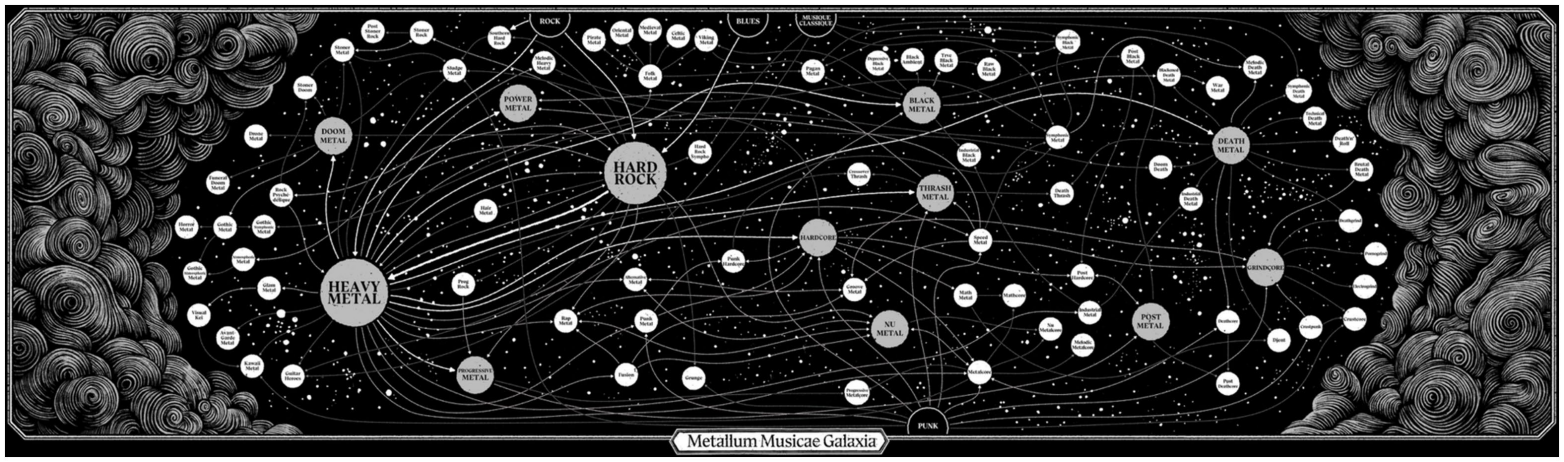
Metallum musicae galaxia, cartography of metal sub-genres.

For the second step, again, a pilot screening was conducted, as recommended in the latest JBI guidelines (Peters et al., 2022), on 10% (*n* = 23) of the identified potential sources (*n* = 230). This helped manage any further disagreements or discrepancies, which were resolved through discussions between the reviewers (C. R. and A. V.) on a Zoom-meeting until consensus was reached.

Considering the potential redundancy of primary sources both individually and as part of literature reviews, this was considered and highlighted during both data extraction and analysis (Peters et al., 2022). Excluded references, along with the reasons for non-inclusion, will be reported as recommended in the JBI guidelines in the PRISMA-ScR flowchart (Peters et al., 2022).

#### Data extraction, analysis and presentation

A pilot data extraction and analysis were conducted on each type of sources identified, as recommended in the JBI guidelines (Peters et al., 2022). Both independent reviewers (C. R. and A. V.) compared their perspective on data extraction and presentation. This process helped manage further disagreements or discrepancies, which were resolved through discussions between the reviewers until consensus was reached. Data extraction consisted in two steps: first, reporting the main information of the paper in a table with five columns: author and date, domain of health, population, study-design and general content. Then, studies were summarised in a few sentences, highlighting the main useful outcomes for this review which could help handle both aforementioned objectives.

Data presentation was performed collaboratively by both reviewers during online meetings.

Finally, following the JBI methodology and PRISMA-ScR checklist (Tricco et al., 2018; Peters et al., 2022), methodological quality assessment was not conducted, as such an appraisal is not required for scoping reviews.

## Results

A total of 5,182 references were identified from the initial literature search. After removing duplicates and inaccessible references, 3,550 references remained for screening. Subsequently, 230 references were retained for full-text assessment, leading to the inclusion of 39 records. Following a manual search in databases and specialised journals (*n* = 8), a total of 47 articles were finally included in this scoping review.

Table 3 describes the included studies in terms of domain, study design, population, and a short summary of the aims or general content of each article.

**Table 3:** Description of the included studies.

Authors, date, location	Domain	Study design	Population (if applicable)	General content
Aaen et al. (2024b); Aaen et al. (2024a)—UK, Denmark & Holland	Vocal health	Cross-sectional study	Professional singers (including metal/rock singers)	This study investigated five vocal effects (distortion, growl, grunt, rattle, and creaking) in terms of acoustics and electroglottography, compared to notes without vocal effects.
Aaen et al. (2024a)—UK, Denmark & Holland	Vocal health	Cross-sectional study	Professional singers (including metal/rock singers)	The authors investigated vocal health of professional singers performing vocal effects (see above) in their daily life—singing and teaching.
Axelsson et al. (1995)—Sweden	Hearing health	Cross-sectional study	Pop/rock musicians	The aim of this study is to investigate thresholds hearing levels in pop/rock musicians 16 years after their first assessment.
Azar (2020)**—Canada	Musculoskeletal and neurological health	Cross-sectional study	Drummers	The study investigates prevalence of musculoskeletal disorders in drummers, including metal drummers.
Bellis et al. (2007)—United Kingdom	Mortality	Retrospective cohort	Rock and pop stars	The authors reported the mortality rates in the All-Time Top 1,000 albums (including rock, punk, rap, R&B, electronica, and new age).
Bodner and Bensimon (2008)—Israel	Mental health	Cohort study	Funk-rock and alternative-rock singers	Singers were asked to fill out questionnaires about their self-esteem, negative and positive effects, and purpose in life before and after performance.
Butkovic and Rancic Dopudj (2017)**—Croatia	Mental health	Cross-sectional study	Heavy metal musicians (compared to classical ones)	This study investigated personality traits in heavy metal musicians, compared to classical ones, in relationship to their alcohol consumption.
Caffier et al. (2018)—Germany	Vocal health	Cross-sectional study	Pop/rock/metal singers (compared to musical theatre ones)	This study aimed to assess potential voice damages while performing vocal effects (breathy, creaky, voice, distortion, grunting, among others).
Carey (2023)—Canada	Mental health	Biographical case report	Per “Dead” Ohlin, member of the Norwegian black metal band Mayhem	The author investigates Dead’s influence 30 years after his suicide on metal music and its artistic implications through the mental health perspective (self-harm, etc.)
Cross et al. (1989)—Australia	Musculoskeletal and neurological health	Conference case report	Rock guitarist	Three experts discussed the case of a 25-year-old guitarist and his knee injury while performing.
Di Stadio et al. (2018)—Italy	Hearing health	Systematic review	Pop/rock musicians (compared to classical ones)	This review aimed to investigate hearing loss, and associated hearing issues in professional musicians, comparing pop/rock to classical musicians.
Drake-Lee (1992)—United Kingdom	Hearing health	Cross-sectional study	Three of the four members of the heavy metal band Manowar	The audiometry was measured and analysed before and half an hour after a concert in three heavy metal musicians.
Egnor et al. (1991)—United States of America	Other	Case report	15-year-old heavy metal musicians	The authors reported the case of a 15-year-old drummer suffering from vertebral artery aneurysm after head banging.
Firle and Richter (2025)—Germany	Hearing health	Scoping review	Professional musicians	This scoping review aimed to investigate both prevalence and characteristics of noise-induced hearing loss in professional musicians.
Gambichler et al. (2004)—Germany	Other	Narrative review	Musical instrumentalists	This narrative review summarised a certain number of instrument-related skin diseases, such as allergies, irritations due to contact, among others.
Güths et al. (2024)—Brazil	Vocal health	Cross-sectional study	Rock singers	Rock singers were asked to perform glottic vocal distortions to analyse anatomical and spectral components.
Guzman et al. (2019)—Chile	Vocal health	Cross-sectional study	Metal singers	The authors investigated aerodynamics when metal singers produced growl voice or reinforced falsetto.
Guzman et al. (2014)—Chile/United States of America	Vocal health	Cross-sectional study	Rock and pop singers	Rock singers (and pop singers for comparison) singing and speaking voice were assessed in terms of acoustic, perceptual, functional and laryngoscopic perspectives.
Hamilton et al. (2019)**—Chile	Mental health	Qualitative study	Heavy metal musicians	Using the phenomenological approach, the authors investigated the concept of flow when considering performance of heavy metal musicians.
Hamilton (2016)—Australia	Mental health	Narrative review	Famous figures who committed suicide	This historical review depicted the suicides of four famous figures, including Kurt Cobain’s.
Hernandez et al. (2009)—United States of America	Mental health	Cross-sectional study	Rock musicians	The authors investigated, using several questionnaires, the psychological profile of a specific rock band, including four members.
Hirosaki et al. (2021)—Japan	Vocal health	Cross-sectional study	Rock singers (compared to classical ones)	This retrospective study aimed to compare vocal fold lesions between rock and classical singers.
Izdebski et al. (2013)	Vocal health	Case series study	Heavy metal singers	Using high-speed digital imaging and distant chip scope, heavy metal growl vocal production was investigated, also in the perspective of vocal health.
Itzkoff (2009)*—United States of America	Musculoskeletal and neurological health	Journal brief article	Steven Tyler, from Aerosmith	The author reported injuries in the band Aerosmith, and more precisely Steven Tyler’s ones, cancelling the tour remaining dates after he fell from the stage during a concert.
Kenny and Asher (2017)**—Australia	Mortality	Retrospective cohort	Popular musicians	This review aimed to investigate mortality and morbidity, considering mainly gender differences, in popular musicians.
Kenny and Asher (2016)—Australia	Mortality	Retrospective cohort	Popular musicians	This review aimed to investigate mortality and morbidity, and more precisely causes of death, in popular musicians.
Kumar et al. (2016)—India	Hearing health	Cross-sectional study	Rock musicians (compared to non-musicians)	This study aimed to explore contralateral suppression of oto acoustic emission, comparing professional rock musicians to non-musicians.
Lee and Altenmuller (2014)—Germany	Musculoskeletal and neurological health	Case report	Heavy metal drummer	Task specific dystonia in the lower limb of a 28-year-old heavy metal drummer is reported and described.
Matsuzaki et al. (2012)—Japan	Other	Case report	Heavy metal guitarist	The authors reported the case of a 34-year-old musician experiencing neck and chest pain, and mediastinal emphysema, after head banging.
McIlvaine et al. (2012)—United States of America	Hearing health	Cross-sectional study	Rock musicians	This study aimed to investigate personal noise dosimetry in professional rock musicians.
Meiling et al. (2022)—United States of America	Other	Scoping review	Music-associated head banging patients	This scoping review aimed to investigate the traumatic brain injuries due to music-associated head banging.
Mula and Trimble (2009)—Italy/United Kingom	Mental health	Narrative review	Musicians in general	This review handles the complex relationship between psychopathology and music, based on famous musicians’ biographies.
Newman et al. (2022)**—United States of America	Mental health	Cross-sectional study	Touring musicians (and their crew)	This paper investigated mental health problems in touring professionals, including artists but also their crew.
Orchard et al. (2024)—Australia	Mortality	Retrospective cohort study	Rock and pop musicians (compared to other populations)	This study investigated the death rates and life expectancy in Australian rock and pop musicians, athletes (football, cricket) and general population.
Patton and McIntosh (2008)—Australia	Musculoskeletal and neurological health	Cross-sectional study	Head bangers	The authors determined traumatic brain and neck injury in head bangers, related to heavy metal music.
Petrescu (2008)—Canada	Hearing health	Narrative review	General population	This review investigated the impact of loud music listening on hearing loss.
Raeburn (2000)**—United States of America	Mental health	Narrative review	Popular musicians	This review, in two parts (1999 and 2000), explored various psychological issues and potential strategies to treat them in popular musicians, illustrating these with famous musicians’ biographical elements.
Raeburn (1999)**—United States of America				
Rigg et al. (2003)—United States of America	Musculoskeletal and neurological health	Cross-sectional study	Guitarists	This study aimed to assessed playing-related injuries in guitarists, and pain locations in relation to the playing posture and movements.
Romero et al. (2016)**—United States of America	Other	Cross-sectional study	Metal drummers	The authors investigated the metabolic demands (oxygen consumption, minute ventilation, heart rate, among other elements) in metal drummers.
Samelli et al. (2012)—Brazil	Hearing health	Cross-sectional study	Pop/rock musicians (compared to non-musicians)	Peripheral and central auditory pathways were investigated in pop/rock musicians compared to non-musicians, to identify how music exposure could impact these components.
Sanches et al. (2022)—Brazil	Mental health	Biographical case report	Per “Dead” Ohlin, member of the Norwegian black metal band Mayhem	The authors describe Ohlin’s behaviours and beliefs in the light of a specific psychiatric condition, the Cotard’s syndrome.
Santoni and Fiorini (2010)—Brazil	Hearing health	Case series study	Pop-rock musicians	This study investigated the degree of satisfaction of pop-rock musicians when using their earing protection devices.
Stormer et al. (2017)—Norway	Mental health	Cross-sectional study	Rock musicians	This study assessed the prevalence of anxiety and depression in rock musicians with or without tinnitus, also considering the influence of this hearing condition on their daily life.
Størmer et al. (2015)—Norway	Hearing health	Cross-sectional study	Rock musicians	This study assessed hearing thresholds and tinnitus (prevalence and characteristics) in rock musicians.
Unterhofer et al. (2025)—Germany	Vocal health	Cross-sectional study	Metal singers	The authors investigated whether if voice disorders (including dysphonia) was more prevalent in autodidact metal singers compared to metal singers who received singing education.
Vellers et al. (2015)—United States of America	Ohter	Cross-sectional study	Professional musicians (including metal ones)	This study investigated heart rate response while playing as an indicator of performance physiological stress in professional musicians, comparing also the effect of genre.

*Not peer-reviewed references. **Articles obtained by searching manually (ISMMS/MPPA). Yellow: vocal health; Orange: hearing health; Green: musculoskeletal and neurological health; Blue: mental health; Purple: mortality; and Grey: Other.

Six categories were developed to classify the included studies:

- Mental health: *n* = 12;
- Hearing health: *n* = 10;
- Musculoskeletal and neurological health: *n* = 9;
- Vocal health: *n* = 9;
- Mortality: *n* = 4;
- Other: *n* = 3.

### Musculoskeletal and neurological health

#### Focal dystonia

Lee and Altenmuller (2014) reported the case of a 28-year-old heavy metal drummer (with pronounced perfectionist traits) presenting with task-specific dystonia in the lower limb, specifically the right thigh. His first symptoms appeared at age 26, when he began to play the drums with a double bass pedal. The main symptoms included “difficulty when playing fast, repetitive tapping patterns with the right foot, and an increasing tightness of the right thigh,” as well as “overflow of EMG activity to the thigh antagonist muscles (quadriceps and biceps) and coactivation.”

#### Instrument-specific disorders

Rigg et al. (2003) investigated playing-related injuries in popular music guitarists. In their sample (*n* = 261), approximately 61% reported playing-related pain in the past year (the authors suspected that guitarists experiencing pain were more likely to participate). The main disorders were located in the fretting hand and wrist (42%), followed by the back (17%) and the neck (15%).

Azar (2020) studied the prevalence and patterns of injuries in 831 drummers (including 37% metal drummers). Within the sample, 68% of participants reported a lifetime occurrence of playing-related musculoskeletal disorders, with the wrist joint (25%) and lumbar spine (24%) being the most frequently affected areas.

#### Head banging

Egnor et al. (1991) described the case of a 15-year-old drummer who developed a traumatic vertebral artery aneurysm caused by head banging while performing with his heavy metal band. The aneurysm occurred 4 weeks after he experienced sudden severe neck pain, vertigo, nausea, and motor and speech disturbances while head banging. The authors suspected the aneurysm was a consequence of violent neck movements, with the first symptoms being due to arterial spasm.

**Figure 2:**
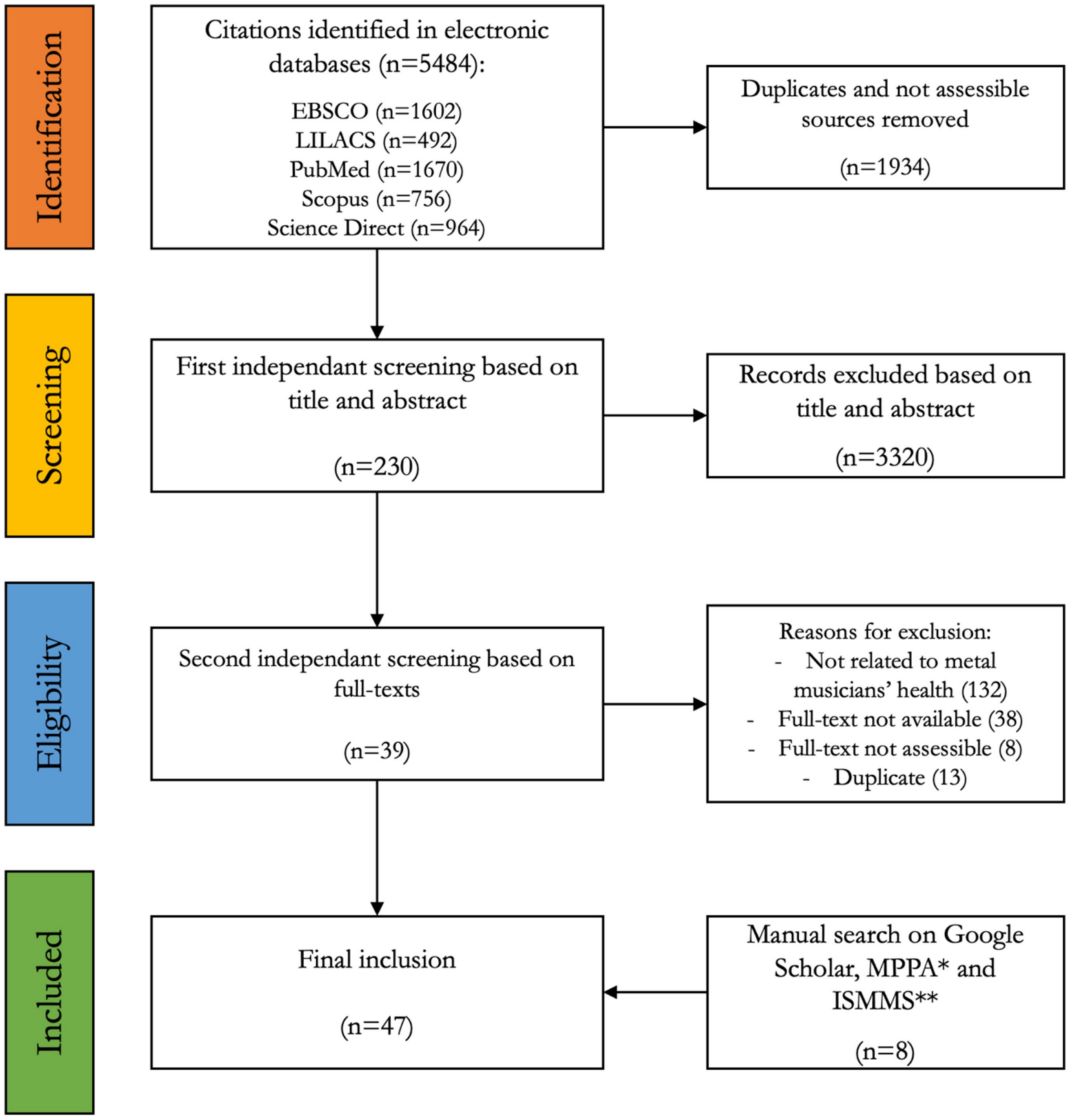
Flow-chart (based on PRISMA-ScR guidelines—Tricco et al., 2018).

Matsuzaki et al. (2012) reported the case of a 34-year-old guitarist diagnosed with mediastinal emphysema after presenting with severe neck pain, which the authors attributed to head banging. They noted similarities with symptoms commonly reported in shaken-baby syndrome (Figure 2).

Patton and McIntosh (2008) investigated mild traumatic brain and neck injuries associated with head banging using a biomechanical model (the Head Injury Criterion), concert observations, and focus groups with head bangers. They described thresholds for head and neck injuries based on tempo (beats per minute) and neck range of motion. Considering the 11 songs chosen by participants, with an average tempo of 146 BPM, mild head injury was predicted when neck movements exceeded 75°.

Meiling et al. (2022) conducted a scoping review on traumatic brain injury related to head banging in a musical context. All included studies were case reports, describing conditions such as traumatic subdural hematoma, internal carotid artery dissection, basilar artery thrombosis, traumatic vertebral artery aneurysm, and intracerebral haemorrhage, all classified as moderate-to-severe traumatic brain injuries. The authors highlighted the absence of studies on mild injuries.

#### Concert injuries

Cross et al. (1989), in a case conference report, described a 25-year-old guitarist who was dragged off stage by fans during a concert and sustained ruptures of both the medial capsular and anterior cruciate ligaments upon landing on his right knee.

In a short article for the New York Times, Itzkoff (2009) reported that Steven Tyler, lead singer of Aerosmith, fractured his shoulder and injured his head after falling from a stage.

#### Conclusion

Included studies and articles discussed about metal musicians’ musculoskeletal health in many ways, which make them difficult to compare. Topics were headbanging, concert injuries, focal dystonia and instrument-specific disorders, such as guitar-related.

### Vocal health

The variables of interest (vocal parameters), measurements, population, and main findings of the studies are summarised in Table 4.

**Table 4:** Description of the included studies focused on vocal health (*n* = 7/9).

Study	Population	Vocal parameters	Measurements	Main findings
Aaen et al. (2024a)	20 singers (10 male, 10 female, 34 year-old, 20–52)—17 singers for the follow-up study (11 for endoscopic re-examination).	Vocal effects (distortion, growl, grunt, rattle, and creaking).	Nasoendoscopic examination and questionnaires (for vocal health) with a 14-year follow-up.	All scores (both from questionnaires and endoscopic) below the usual scores for healthy singers. No detriment to vocal health related to vocal effects.
Aaen et al. (2024a); Aaen et al. (2024b)	32 healthy professional singers (20 female, 12 male, 45 year-old, 31–65).	Four vocal modes (neutral, curbing, overdrive, edge) with/without five vocal effects (distortion, growl, grunt, rattle, and creaking).	Electroglottography and long-term average spectrum for acoustics.	Most effect are associated with supraglottic structures movements, without using vocal folds, leading to no detriment to vocal health (except for some cases of grunt and creaking cases).
Caffier et al. (2018)	10 male singers (34 years old, 25–46)—background: musical theatre (Azar, 2020), pop/rock/metal (Azar, 2020)	Different vocal effects: breathy voice, creaking, vocal fry, grunt, distortion, rattle, belt, and twang.	Videolaryngoscopy, videostroboky-mography, transcutaneous electroglottography, acoustic-aerodynamic diagnostics, auditory-perceptual assessment.	The vocal apparatus was healthy in all singers, despite long years of singing. No mechanical damage was observed on vocal folds nor laryngeal mucosa. Partial glottal vibration was outlined as a potential cause of loudness loss in singers.
Güths et al. (2024)	3 males singers using glottic vocal distortions (mean age: 31 years old).	Singing voice with and without vocal disortions.	Auditory-perceptual assessment, nasolaryngospic and larynx funciontal assessments.	Vocal distortions can be performed without leading to damage to the vocal apparatus.
Guzman et al. (2014)	21 rock (19 male, 2 female; mean age: 26 years old, 19–34) and 18 control pop singers.	Speaking and singing voice, including “eddy voice,” i.e., growl voice and reinforced falsetto.	Acoustic, perceptual, functional and laryngoscopic assessments.	Growling and reinforced falsetto induce supraglottic compression, pharyngeal constriction and changes in vertical laryngeal position, which do not cause laryngeal disorders in the sample of rock singers.
Guzman et al. (2019)	54 metal singers	Growl voice and reinforced falsetto.	Laryngeal videostroboscopy first to assess the absence of pathology; aerodynamic and acoustic measurements.	Decrease of vocal folds adduction during growl voice and increase of subglottic pressure; increase of both during reinforced falsetto. Reduction of glottal adduction and better strategy for voice resonance might prevent voice problems in singers.
Izdebski et al. (2013)	An experienced heavy metal singer.	Heavy metal growl.	High speed digital imaging and distant chip scope for visual examination.	The analysis showed supraglottic vibrations with occasional adduction of the vocal folds, and an absence of glottic closure, which might both prevent lesions of the vocal folds.

Hirosaki et al. (2021) presented retrospective data from singers with benign vocal fold lesions who underwent laryngeal surgery. Their sample included 36 rock (22 male, 14 female) and 24 classical (11 male, 13 female) professional singers. The authors reported that lesions were more common in classical singers compared to rock singers, suggesting that sex (females reporting more upper lesions, and males more lower lesions), vocal register (classical females using more head voice compared to others using modal voice), and therefore mechanical stress on vocal folds may influence lesion location.

Unterhofer et al. (2025) investigated the experiences of metal singers regarding their vocal training and health. In their sample (*n* = 74), 12.2% reported dysphonia, but no dysphonia was found in full-time professional singers or singers with formal vocal training. No significant correlations were observed between several potential risk factors (such as vocal training, recovery time, sex, age, or alcohol/tobacco use) and the presence of dysphonia.

#### Conclusion

The included studies consistently confirmed that the use of the ‘false’ vocal folds might be protective for vocal health, and highlighted a weak prevalence of dysphonia and less lesions compared to classical singers.

### Hearing health

#### Reviews

The narrative review from Petrescu (2008), in a narrative review, reported that rock musicians face a substantial risk of hearing disorders, including hearing loss, tinnitus, and hyperacusis. The author also noted limited evidence that hearing protection significantly reduces damage.

More recently, Firle and Richter (2025) conducted a scoping review on hearing loss in musicians. Rock musicians were not separated from pop and jazz musicians, but the literature indicated that hearing loss is frequent among these musicians in the 3,000–8,000 Hz frequency range, and more common than in classical musicians.

Finally, Di Stadio et al. (2018) compared pop/rock musicians to classical musicians in a systematic review of hearing disorders. They found that pop/rock musicians were at greater risk for hearing loss and hyperacusis, while tinnitus, the most reported disorder, affected both groups equally.

#### Clinical trials

Axelsson et al. (1995) conducted a 16-year follow-up study on hearing health in pop/rock musicians. In their sample (*n* = 40), 78% were considered to have normal (or near normal) hearing, with thresholds improving compared to the first assessment. Nevertheless, more than 65% reported ear-related complaints, and percussionists were the most affected. The authors noted they were unable to fully explain the apparent hearing resilience in musicians.

Drake-Lee (1992) assessed audiometry in three male musicians (25–37 years old) from a heavy metal band during their international tour, before and after a concert. Temporary threshold shifts (particularly at lower frequencies) were reported in all unprotected ears.

Kumar et al. (2016) investigated contralateral suppression of otoacoustic emissions in rock musicians, a mechanism described as protective. They reported significantly greater suppression at 1 kHz in musicians compared to non-musicians (*p* < 0.05), but not at other frequencies, suggesting enhanced protection against prolonged exposure.

McIlvaine et al. (2012) measured noise exposure in male rock band members (40–60 years old) during rehearsals and performances using dosimeters and compared outcomes to industry guidelines. They found dangerously high sound levels putting musicians at risk of hearing loss, necessitating the use of protective devices.

Samelli et al. (2012) evaluated both peripheral and central auditory pathways in male pop/rock musicians (18–45 years old) compared to non-musicians. Musicians showed worse hearing thresholds and lower transient evoked otoacoustic emission amplitudes, indicating cochlear damage and risk of hearing loss. However, shorter ABR and P300 latencies suggested enhanced central auditory processing, likely due to musical training.

Santoni and Fiorini (2010) assessed satisfaction among pop-rock musicians (males, 25–45 years old) using hearing protectors over 3 months. They reported a high prevalence of hearing loss (about 22%), and positive correlations between hearing protector use and tinnitus complaints, although musicians reported some dissatisfaction (e.g., ear pressure, autophonia).

Størmer et al. (2015) investigated the prevalence of hearing loss and tinnitus in Norwegian rock musicians (102 males, 15 females, 16–52 years old). They found that 37.8% [95% CI: 28.8–46.8%] had hearing loss, particularly at 6 kHz, with singers most affected. Unexpectedly, the authors also reported lower thresholds inversely associated with noise exposure.

#### Conclusion

Hearing health was highlighted as a matter of importance in metal musicians, as in all musicians, including orchestra ones. The described disorders were tinnitus or hearing loss, and protective strategies were also discussed.

### Mental health

#### Biographical analysis

Per “Dead” Ohlin, member of the Norwegian black metal group Mayhem (1988–1991), who died by suicide at age 22, and his mental health disorders were analysed in two papers. Sanches et al. (2022) discussed a possible post-mortem psychiatric diagnosis of Cotard’s syndrome, a rare depressive condition. They highlighted how the specific context of black metal, with its themes of death worship and anti-Christianism, may have masked Ohlin’s psychiatric condition, as well as the lack of evidence regarding psychiatric care. Carey (2023) examined Ohlin’s artistic legacy in light of his mental illness, focusing on self-inflicted violence and pain in performance. They discussed the influence of his self-destructive acts on black metal culture, while cautioning against glorification.

#### General reviews

Raeburn provided a two-part review on popular musicians and mental health (Raeburn, 2000; Raeburn, 1999). The first part described psychological and creative development, highlighting risk factors such as family environment, community, and occupational stressors (e.g., band roles, conflicts, job status, career development). Raeburn distinguished four categories of musicians, as aspiring, working, celebrity, and “superstar,” each with specific challenges. The second part (2000) described health issues such as depression, anxiety (including performance anxiety), substance abuse, and dependence, also in relation to creativity. Treatment strategies could be individually tailored but may also involve “treating the band” and the wider music industry.

Mula and Trimble (2009) discussed the complex relationship between psychiatric conditions and musical creativity, illustrated with famous cases from classical, jazz, and rock musicians. In their conclusion, they highlighted mental health issues (self-harm, suicidal ideation, depression, drug use) in relation to rock/metal music preferences, citing Kurt Cobain’s suicide as an example of the “Werther effect,” and discussed suicide themes in lyrics.

Hamilton (2016) analysed four high-profile suicides, including that of Kurt Cobain, who shot himself in April 1994. The author reported Cobain’s severe depression and public acknowledgment of his mental health struggles, including suicidal ideation. The potential Werther effect of his suicide was also discussed, given his immense celebrity and possible impact on public health.

#### Epidemiological studies

Bodner and Bensimon (2008) investigated self-esteem, affect, and purpose in life in professional rock singers before and 3 days after a performance. All scores except self-esteem were significantly higher before the performance. The authors noted that their findings aligned with previous research showing frequent psychological issues among performing artists.

Butkovic and Rancic Dopudj (2017) examined alcohol consumption and personality traits (Big Five taxonomy) in classical and heavy metal musicians. Metal musicians reported significantly higher alcohol consumption (*p* < 0.05) compared to both classical musicians and the general population. No significant differences were found in personality traits between groups, but all musicians scored higher than the general population on extraversion, agreeableness, and intellect (*p* < 0.01).

Hernandez et al. (2009) analysed the “psychological profile” of four rock band members using intellectual and personality assessments. They reported difficulties such as coping with daily life, denial of mental or physical health disorders, and impulsive tendencies potentially affecting band functioning.

Hamilton et al. (2019), from a phenomenological perspective, investigated flow in metal musicians. They found that flow was enabled by criteria such as immediate feedback, clear goals, concentration, focus, control, awareness, and autotelic experience, but also influenced by emotions (e.g., anger), relationships (within the band and with the audience), and even clothing.

Newman et al. (2022) assessed mental health in a large sample of touring professionals. Both artists and crew members reported numerous stressors affecting their mental health. Compared to the general population, participants showed mild anxiety scores (classifying them at risk), high rates of depressive predisposition (50% vs. 4%), and high suicidality (40% vs. 8%), among other concerning findings.

Stormer et al. (2017) investigated anxiety and depression in musicians with hearing disorders (loss or tinnitus). Compared to controls, rock musicians were at risk for permanent tinnitus (19.8% vs. 0%) and more frequently reported anxiety symptoms (35.1% vs. 17.5%, *p* < 0.05). Depression scores were also higher among tinnitus-affected musicians (*p* < 0.05).

#### Conclusion

The heterogeneity of the included studies about mental health makes them difficult to compare. Several factors, such as touring or auditory disorders, were outlined as potentially leading to anxiety or depression. Biographical cases were also included, and other mental health components, such as self-esteem or substance abuse were mentioned.

### Mortality

Bellis et al. (2007) identified solo performers and band members from the All-time Top 1,000 Albums list and analysed their biographies to determine survival status and causes of death by the end of 2005. Their sample included nearly 90% rock musicians and 4% punk musicians. Among 1,064 cases across Europe and North America, the main causes of death were cancer (20%), drug/alcohol overdoses (19% vs. 15% in North America and 29% in Europe), and cardiovascular disease (14% vs. 3.6% in Europe and 18% in North America). Mortality in pop stars exceeded that of the general population before reaching 25 years of fame.

Kenny and Asher (2016, 2017) defined popular music as including all forms of rock and metal. In their 2016 paper, they compared male deaths before 65 years of age and found significantly higher rates among metal musicians compared to the general population, due to accidents (including overdoses: 259 deaths vs. 167 expected, *p* < 0.001), suicide (137 vs. 68, *p* < 0.001), liver-related disease (34 vs. 16, *p* < 0.001), respiratory disease (12 vs. 5, *p* < 0.001), and cancer (138 vs. 76, *p* < 0.001). Results were similar for rock musicians. In their 2017 paper, Kenny & Asher focused on gender differences. Among female metal musicians dying before 45, only violent deaths (accidents, homicide, suicide) were higher than expected (15 vs. 8, *p* < 0.05). Among male metal musicians dying before 45, violent deaths (405 vs. 349, *p* < 0.05), liver-related causes (23 vs. 11, *p* < 0.05), and cancer (88 vs. 53, *p* < 0.05) were higher. Among male musicians dying after 45, only violent deaths were higher (41 vs. 20, *p* < 0.05).

Orchard et al. (2024) compiled biographies of Australian rock and pop solo musicians (*n* = 655, Wikipedia-based) and listed causes of death as cancer, cardiovascular disease, injury/violence (including drug overdose), and other causes. They compared standardised mortality rates (SMR) across musicians, athletes, and the general population. Musicians had higher SMR than athletes (SMR 1.35 [95% CI 1.07–1.71] for rock/pop musicians vs. SMR 0.77 [95% CI 0.74–0.80] for footballers) and the general population, with particularly elevated mortality in women and from cancer.

### Other

Gambichler et al. (2004) listed a range of skin disorders in instrumental musicians. Among guitarists and drummers, which are common instruments in metal music, possible conditions include hand callosities in drummers (“drummer’s digit”), nickel or chromium allergies in guitarists (“guitar nipple”), as well as instrument-related infections or miscellaneous disorders such as “guitar groin,” acro-osteolysis, or hyperhidrosis.

Vellers et al. (2015) investigated heart rate in rock musicians to assess physiological stress while playing. The overall mean maximum heart rate during rehearsals (52 ± 5%) and performances (59 ± 5%) matched values for moderate physical exercise, suggesting that playing music is physiologically demanding. No significant differences were observed between rehearsal and performance (*p* = 0.40), except for classic rock musicians, where higher rates were noted (*p* < 0.05).

Romero et al. (2016) monitored drum performance in five drummers, highlighting that metal drumming, due to its energy demands, can be considered a vigorous physical activity, with drummers’ VO₂ peak values reaching up to 90% of their VO₂max when playing at the highest tempi.

## Discussion

This scoping review is, to the best of our knowledge, the first to show an interest and synthesise available evidence on both the physical and mental health of musicians who play metal music. As many studies did not focus exclusively on metal musicians but also included rock or pop/rock performers, the scope of the review extended beyond metal alone. This is justified, however, as these musicians often share common risk factors, such as touring, exposure to high levels of music-related noise, or instrument-related physical demands. Nevertheless, the analysis of the 47 included studies highlights significant gaps in the literature, as well as specific challenges associated with the context of heavy metal music. Six thematic categories emerged: musculoskeletal, vocal, hearing and mental health, life expectancy and mortality, and other aspects of health.

### Considerations about physical health

Musculoskeletal and neurological health issues were diverse across instrumentalists, whether they were metal or rock musicians. These performers share risk factors with musicians from other genres, such as instrument-specific postural loads or repetitive movements, which can contribute to musculoskeletal and neurological disorders (Rigg et al., 2003; Lee and Altenmuller, 2014). However, headbanging appears to be a practice specific to metal. In the studies included here, headbanging was associated with conditions ranging from cervical strain to severe intracranial injury (Meiling et al., 2022; Patton and McIntosh, 2008). Rare case reports also described vertebral artery aneurysm (Egnor et al., 1991) and mediastinal emphysema (Matsuzaki et al., 2012). Finally, drummers in heavy metal can be considered physical athletes, as their energy expenditure reaches levels comparable to those observed in vigorous physical activities (Romero et al., 2016).

Vocal health is another key area of concern. Contrary to common assumptions, the use of vocal effects such as growling, grunting or creaking has not been shown to cause structural vocal damage in cross-sectional studies (Aaen et al. 2024a; Caffier et al., 2018; Guzman et al., 2019). Some evidence even suggests that supraglottic involvement may protect the vocal folds (Izdebski et al., 2013). Nonetheless, longitudinal studies are needed to determine long-term risks, including vocal fatigue and potential technical impairments.

**Figure 3:**
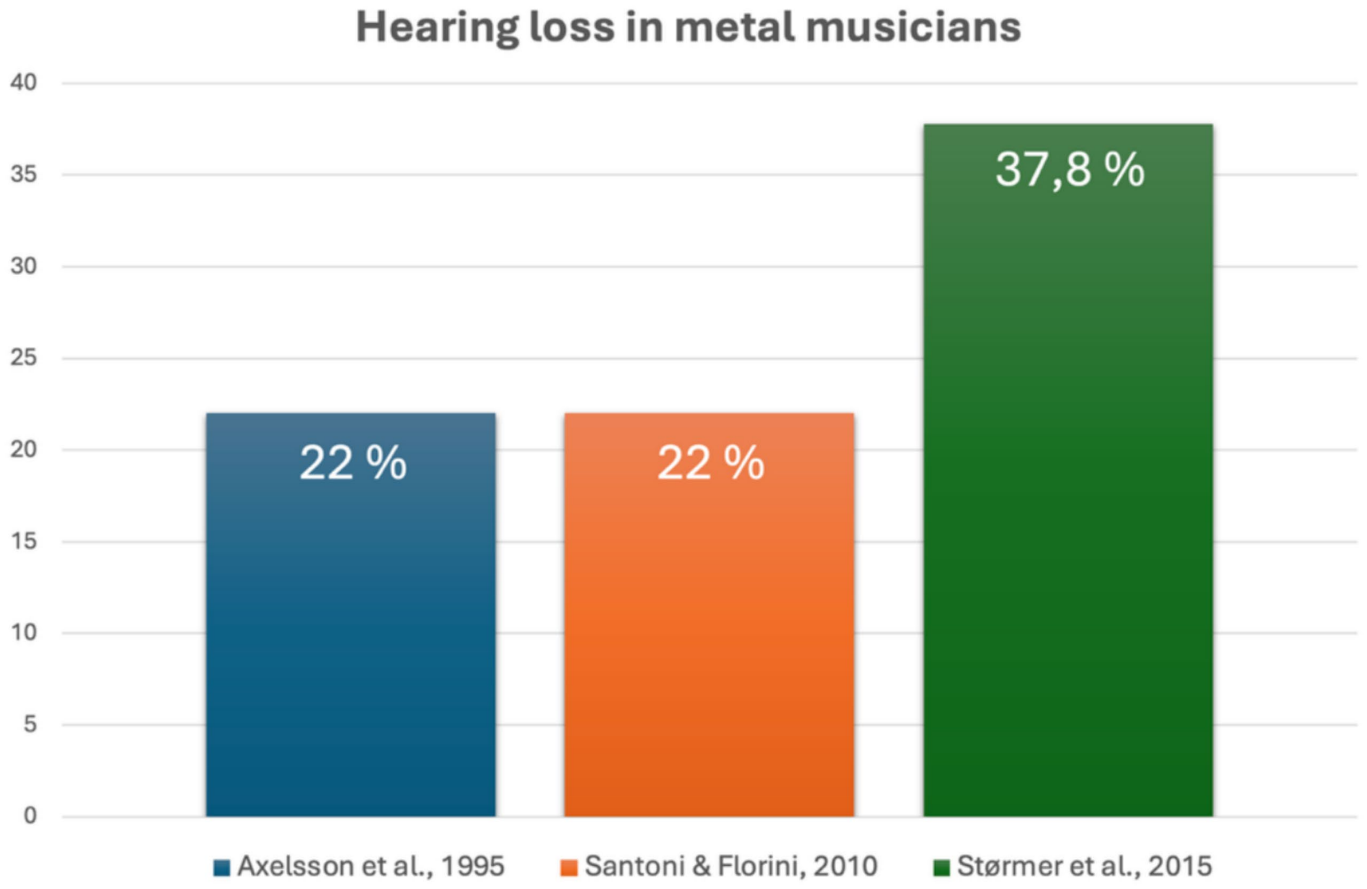
Graphical comparison of three studies investigating hearing loss in metal musicians.

Hearing health was also frequently investigated. Metal (and rock) musicians reported hearing loss, tinnitus, and hyperacusis (Størmer et al., 2015; Di Stadio et al., 2018). This is unsurprising given the high sound exposure inherent to their profession (McIlvaine et al., 2012), even though some musicians reported using hearing protection (Santoni and Fiorini, 2010). Figure 3 compares percentage of reported hearing loss in three studies (Axelsson et al., 1995; Santoni and Fiorini, 2010; Størmer et al., 2015).

Initially, this review aimed to map both prevalence and risk factors among metal musicians, and if some factors appeared clearly, such as physically and mentally demanding tours, specific movements on stage, or high-volume music performances, it was not possible to determine the existence of other factors related to musculoskeletal health, nor the prevalence of possible performance-related health conditions.

### Considerations about mental health and other aspects

Mental health outcomes proved difficult to summarise due to the heterogeneity of sources, which ranged from biographical reports to small empirical studies. Some articles emphasised individual vulnerability, such as the possible Cotard’s syndrome diagnosis of Per “Dead” Ohlin (Sanches et al., 2022) or the widely discussed example of Kurt Cobain’s suicide (Hamilton, 2016). Others focused on mortality rates in rock and metal musicians, revealing higher-than-expected rates of premature death, particularly from violent causes such as accidents, overdoses, and suicides (Kenny and Asher, 2016, 2017).

Broader occupational stressors were also reported. Touring, for example, emerged as a major factor affecting musicians’ mental health (Newman et al., 2022). On the other hand, some studies explored positive aspects, such as the role of flow in metal musicians, which is considered central to artistic motivation (Hamilton et al., 2019). Overall, findings in this domain must be interpreted with caution to avoid simplistic associations between extreme music genres and psychopathology.

### Being a metal musician: is that really different?

This review sought to investigate the specific stressors and health conditions faced by metal musicians. While certain practices, such as headbanging, appear unique, further research is needed to determine how these specificities translate into distinct health outcomes. Importantly, performing music is not always the sole occupation for metal musicians. As Raeburn (1999) outlined for popular musicians more generally, four categories can be distinguished: aspiring, working, celebrity, and superstar musicians. Health impacts likely vary across these groups. For instance, professional singers with “day jobs” requiring vocal use may experience cumulative strain (Bartlett and Wilson, 2017), whereas internationally renowned artists who spend extensive time touring may be at greater risk of mental health problems (Newman et al., 2022).

During the screening process, numerous references were identified regarding the social construction of metal music fans, raising relevant considerations for musicians as well. Heavy metal functions as a cultural framework, with symbols such as long hair and distinctive clothing serving as markers of belonging (Larsson, 2013). Hines and McFerran (2014), studying metalheads retrospectively, underscored the importance of engagement with metal music during adolescence, particularly in relation to emotion regulation, identity development, and social connectedness. These findings caution against simplistic associations between listening to metal and mental health vulnerability. Similar to Pavez et al. (2021), who analysed themes of madness in Spanish punk music, future studies might also investigate lyrical content in metal and its potential relationship to health.

### Strengths, limitations and perspectives

Overall, this review demonstrates both the parallels between health issues in metal musicians and those in musicians of other genres, as well as the unique challenges posed by the technical, cultural, and performance demands of metal music, such as headbanging and other specific music-related movements. However, most contributions were case reports, case series, or small cross-sectional studies, and this review identifies a clear lack of longitudinal studies and of prevalence studies which might improve our knowledge about metal musicians’ health and factors which could interfere with their health conditions, and their musical performance. This review also highlights that several health domains are scarcely investigated such as cardio-vascular health or skin problems, as well as musculoskeletal disorders, which might often occur in these musicians, who perform highly repetitive movements in awkward postures and demanding ones.

Moreover, the scoping review methodology inherently limits the ability to generate practice-oriented recommendations (Munn et al., 2018). Also, the protocol of this review was not fully registered, which can be considered as a limitation for this study.

Future research should employ more robust epidemiological and longitudinal designs to clarify prevalence rates, risk factors, and causal pathways. This would improve the evidence base for prevention and intervention strategies. Cultural aspects, such as identity construction and the social meanings of metal, also warrant greater integration into health research on musicians (Hines and McFerran, 2014; Larsson, 2013), and these elements could be explored using qualitative research designs. From a biomechanical perspective, further investigation of headbanging could inform the development of motor control or strengthening programmes to support safer performance practices. To investigate better potential relationships between factors such as headbanging or repetitive movements with musculoskeletal injuries, prospective cohort studies are needed.

## Conclusion

This scoping review provides the first comprehensive synthesis of evidence on the physical and mental health of metal musicians. While they share many stressors with musicians from other genres, they also face unique challenges due to the specific technical and performative demands of metal, including auxiliary movements (e.g., headbanging), extreme vocal effects, and instrumental skills. These elements differentiate metal musicians from other performers and highlight the need for tailored research and prevention strategies.

Further studies with larger samples and longitudinal designs are required to improve understanding of this population, while also considering the broader cultural context and the role of heavy metal in socialisation. The findings underscore the need for interdisciplinary, culturally informed, and clinically relevant approaches to research in this area.

## Data Availability

The original contributions presented in the study are included in the article/supplementary material, further inquiries can be directed to the corresponding author.
